# *Methylobacterium* sp. 2A Is a Plant Growth-Promoting Rhizobacteria That Has the Potential to Improve Potato Crop Yield Under Adverse Conditions

**DOI:** 10.3389/fpls.2020.00071

**Published:** 2020-02-14

**Authors:** Cecilia Eugenia María Grossi, Elisa Fantino, Federico Serral, Myriam Sara Zawoznik, Darío Augusto Fernandez Do Porto, Rita María Ulloa

**Affiliations:** ^1^ Laboratorio de Transducción de Señales en Plantas, Instituto de Investigaciones en Ingeniería Genética y Biología Molecular (INGEBI), Consejo Nacional de Investigaciones Científicas y Técnicas (CONICET), Ciudad Autónoma de Buenos Aires, Argentina; ^2^ Plataforma de Bioinformática Argentina, Instituto de Cálculo, Ciudad Universitaria, Facultad de Ciencias Exactas y Naturales, Universidad de Buenos Aires (UBA), Ciudad Autónoma de Buenos Aires, Argentina; ^3^ Cátedra de Química Biológica Vegetal, Departamento de Química Biológica, Facultad de Farmacia y Bioquímica, Universidad de Buenos Aires (UBA), Ciudad Autónoma de Buenos Aires, Argentina; ^4^ Departamento de Química Biológica, Universidad de Buenos Aires (UBA), Ciudad Autónoma de Buenos Aires, Argentina

**Keywords:** *Methylobacterium*, plant growth-promoting rhizobacteria (PGPR), salt stress, *Phytophthora infestans*, potato

## Abstract

A Gram-negative pink-pigmented bacillus (named 2A) was isolated from *Solanum tuberosum* L. cv. Desirée plants that were strikingly more developed, presented increased root hair density, and higher biomass than other potato lines of the same age. The 16S ribosomal DNA sequence, used for comparative gene sequence analysis, indicated that strain 2A belongs to the genus *Methylobacterium*. Nucleotide identity between *Methylobacterium* sp. 2A sequenced genome and the rest of the species that belong to the genus suggested that this species has not been described so far. *In vitro,* potato plants inoculated with *Methylobacterium* sp. 2A had a better performance when grown under 50 mM NaCl or when infected with *Phytophthora infestans*. We inoculated *Methylobacterium* sp. 2A in *Arabidopsis thaliana* roots and exposed these plants to salt stress (75 mM NaCl). *Methylobacterium* sp. 2A-inoculated plants, grown in control or salt stress conditions, displayed a higher density of lateral roots (p < 0.05) compared to noninoculated plants. Moreover, under salt stress, they presented a higher number of leaves and larger rosette diameter. In dual confrontation assays, *Methylobacterium* sp. 2A displayed biocontrol activity against *P. infestans*, *Botrytis cinerea*, and *Fusarium graminearum*, but not against *Rhizoctonia solani*, and *Pythium dissotocum*. In addition, we observed that *Methylobacterium* sp. 2A diminished the size of necrotic lesions and reduced chlorosis when greenhouse potato plants were infected with *P. infestans*. *Methylobacterium* sp. 2A produces indole acetic acid, solubilizes mineral phosphate and is able to grow in a N_2_ free medium. Whole-genome sequencing revealed metabolic pathways associated with its plant growth promoter capacity. Our results suggest that *Methylobacterium* sp. 2A is a plant growth-promoting rhizobacteria (PGPR) that can alleviate salt stress, and restricts *P. infestans* infection in potato plants, emerging as a potential strategy to improve crop management.

## Introduction

In 30 years, the world population will be close to 10 billion people, there will be a further 2–3 billion people to feed. According to FAO Statistical Database^1^, 50% of the habitable land is nowadays used for agriculture, most of which is used for the rearing of livestock and only 23% (11 million km^2^) is for food crop production. These 11 million km^2^ supply more calories and proteins for the global population than the almost four-time larger area devoted to livestock (Our World in Data^2^). Today, our challenge is to increase crop productivity at a faster rate than population growth. Most countries have managed to achieve this goal in recent decades; a combination of agricultural technologies, irrigation, improved crop varieties, fertilizers, and pesticides, were used to obtain higher yields (Roser and Ritchie, 2017). However, it is possible that yield gains in the decades to follow will be offset by a growing population. In the near future, especially in the developing world, there will be an increasing prevalence of farming on marginal, arid, and semiarid lands (Coleman-Derr and Tringe, 2014). Therefore, crop yield has to be further improved and crop varieties should be adapted to hostile environments.

Potato is produced in over 100 countries and is the third most important food crop in the world after rice and wheat in terms of human consumption. One hectare (ha) of potato can yield two to four times the food quantity of grain crops; potatoes produce more food per unit of water than any other major crop and are up to seven times more efficient in using water than cereals (CIP International Potato Center^3^). From 1994 to 2014, productivity gains in potato increased 15 points above the population growth rate (Our World in Data^2^). In Argentina, 70 to 80 thousand ha are allocated to potato production, yield values are around 30 to 35 TN/ha, and total production is 2.1 to 2.5 million TNs per year (Garzón and Young, 2016). However, it was estimated that potato productive yield is only 40% to 76% or 47% to 81% of the potential yield, depending on the method used to estimate potential yield, the year and the region (Cantos de Ruiz et al., 1989; de la Casa et al., 2014).

Potential yields can be calculated assuming that there are no abiotic limitations to the growth and there are no biotic factors that reduce growth. The potato plant is well adapted to a number of environmental conditions but certain biotic and abiotic stresses cause significant reductions in growth and yield. Among abiotic stresses, salinity is one of the main constraints that limit plant productivity and cause loss of arable land (Isayenkov and Maathuis, 2019). In particular, potato plants are glycophytes moderately sensitive to salt stress. Although there are differences between cultivars, all cultivars showed a reduction in shoot length, reduced root system development, and reduced tuber yield due to salinity (Dahal et al., 2019). Among biotic stresses, late blight of potato caused by the hemibiotrophic oomycete *Phytophthora infestans* is still a devastating disease worldwide. Chemical management is a popular strategy to control late blight but it may have a negative impact on the environment (Lal et al., 2018).

Numerous techniques have been used to understand the mechanisms and provide tools to enhance plant tolerance under environmental stresses. To ensure long-term food production, we must develop sustainable agricultural practices with minimal adverse impact on the environment. In this context, the use of microbial inoculants plays a key role. When introduced to seeds, roots or into the soil, plant growth-promoting rhizobacteria (PGPR) can solubilize insoluble phosphates, produce plant growth hormones, convert atmospheric nitrogen to ammonia or suppress the growth of plant pathogens (Pérez-Montaño et al., 2014). Naturally occurring PGPR were shown to be effective in enhancing plant growth and development, and in promoting crop productivity and disease management under stress conditions (García et al., 2017; Singh and Jha, 2017; Cordero et al., 2018). This environmentally friendly approach could be among the most efficient methods for minimizing the use of chemicals.

In this work, we have identified and characterized *Methylobacterium* sp. 2A, a PGPR that promotes plant growth and is able to mitigate the harsh effect of salinity on *in vitro* potato and Arabidopsis plants. Moreover, it can reduce *P. infestans* infection on potato greenhouse plants. Genome sequencing allowed us to identify genes that could be involved in its plant growth-promoting (PGP) capacity. We confirmed that this rhizobial can produce indole acetic acid (IAA), is able to grow in N_2_ depleted media, and can solubilize inorganic phosphate.

## Materials and Methods

### Plant and Pathogen Material

*Solanum tuberosum* L. cv. Desirée wild-type (WT) and *Arabidopsis thaliana* Columbia (Col-0) plants were used. Fresh internodes (the first three from the top) from 1-month-old virus-free potato plants were micropropagated in MS medium (Murashige and Skoog, 1962) with the addition of 2% (w/v) sucrose and 0.7% (w/v) agar. Surface disinfected Arabidopsis seeds were vernalized for two days at 4°C and, were then allowed to grow in MS medium (0.5X, 0.8% (w/v) agar). Plants were grown for the indicated times in a growth chamber under a 16 h light photoperiod at 21°C–23°C.

Certified pathogen tested *Solanum tuberosum*, L. var. Spunta (Diagnósticos Vegetales S.A., Mar del Plata, Argentina) tubers were planted in 1-L pots filled with MultiProTM substrate (GrowMix^®^, Terrafertil S.A., Bs. As., Argentina). Soil-grown plants were cultivated in a greenhouse under a 16-h light photoperiod; natural light was supplemented by sodium lamps providing 100–300 µmols^-1^m^-2^; the temperature was set at 25°C during the day and 20°C in the night.

*P. infestans* isolate Pi-60 was kindly provided by Dr. Natalia Norero (Laboratorio de Agrobiotecnología, INTA EEA-Balcarce), maintained on rye sucrose agar (RSA) medium at 19°C ± 1°C in the dark. Suspensions of *Phytophthora* zoospores were performed as described in Fantino et al. (2017). The concentration was adjusted to 25 sporangia µl^-1^ to be used as an inoculum. The fungi *Fusarium graminearum, Botrytis cinerea, Phytium dissotocum,* and *Rhizoctonia solani* were maintained in potato dextrose agar (PDA) medium at room temperature in the dark, or at 19°C ± 1°C in the case of *P. dissotocum*.

### Isolation and Identification of *Methylobacterium* sp. 2A

Bacterial isolation was conducted from roots of potato plants established *in vitro* that were previously grown in the greenhouse at Instituto de Investigaciones en Ingeniería Genética y Biología Molecular (INGEBI-CONICET, Argentina; 34° 33' 28.0” S 58° 27' 32.0” W). Fresh root samples were collected, surface sterilized, and placed in LB agar (Luria-Bertani) at 28°C for 4 days. Pink-pigmented colonies were selected and the isolate was maintained on LB medium without the addition of NaCl (LBNS). To determine the optimal growth conditions, different temperatures (18°C, 24°C, 28°C, 30°C, and 37°C) and culture media (LBNS, PDA, pea sucrose agar (PSA), and RSA) were tested. pH tolerance was determined in LB adjusted to different pH values (4.5 to 7.5). A catalase slide test was performed and oxidase activity was determined by the oxidation of discs impregnated with N, N-dimethyl-p-phenylenediamine oxalate (Britania S.A., Argentina). Antibiotic resistance to streptomycin, hygromycin, rifampicin, ampicillin, kanamycin, chloramphenicol, and cefotaxime were tested in liquid LBNS.

### *16s Ribosomal DNA* Gene-Based Analyses

Genomic DNA (gDNA) was extracted manually according to Chen and Kuo (1993) from a 48-h culture. The 16S ribosomal DNA (*16S rDNA*) gene was amplified by Polymerase Chain Reaction (PCR) with universal primers fD1 and rP2 (**Table S1**) (Genbiotech, Buenos Aires, Argentina) (Weisburg et al., 1991) and sequenced at Macrogen Sequencing facility (Seoul, South Korea). Phylogenetic analysis was performed comparing *Methylobacterium* sp. 2A *16S rDNA* against representative type species of the *Methylobacterium* genus, and against eleven species that have been recently reclassified into the new *Methylorubrum* genus (Green and Ardley, 2018). Multiple sequence alignment and phylogenetic tree reconstruction were performed with MEGA7 (Molecular Evolutionary Genetics Analysis 7, Kumar et al., 2016) software using the Maximum Likelihood method based on the Kimura two-parameter model with 1,000 bootstrap values. Members of the Methylocystaceae family were included as outgroups. Sequence similarity was also compared to those on the EzBioCloud^3^ database (Yoon et al., 2017).

### Plant Inoculation With *Methylobacterium* sp. 2A and Salt Treatments

*In vitro* potato plants were inoculated with *Methylobacterium* sp. 2A by root contact; plant internodes of potato plants showing root pink-pigmentation were equidistantly placed in flasks containing solid MS media with noninoculated potato internodes. Bacterial colonization was evident to the naked-eye two weeks later.

Internodes from potato plants were grown in solid MS media with the addition or not (controls) of increasing NaCl concentrations for 21 days; root and shoot length (cm), and total chlorophyll content (Hiscox and Israelstam, 1979) were assessed. Significant stress was evidenced at 50 mM, but it was still possible to determine chlorophyll content and root growth (**Figure S1**). Therefore, this concentration was chosen to perform further studies. Internodes from potato plants, inoculated or not with *Methylobacterium* sp. 2A, were grown for 21 days in solid MS media with the addition or not of 50 mM NaCl. The experiment was performed three times, using two flasks with four plants each, for each condition (control, C; inoculated, I; salt stress, S; inoculated plants with salt stress, I+S). Root and shoot length, the number of leaves and chlorophyll content were determined at the time of harvest in both control and salt treatments. When measuring root and shoot length, the original internode was not considered. Chlorophyll content was measured on fully expanded leaves as SPAD units using a portable chlorophyll spectrophotometer (Clorofilio^®^, Cavadevices, Argentina).

One-week-old Arabidopsis plants with similar growth were selected and established in Petri dishes containing MS 0.5X, or MS 0.5X with 75 mM NaCl (salt stress conditions; Ruiz Carrasco et al., 2007). After 48 h, plants were inoculated with 2 µl of a sterile saline solution (0.85% NaCl) or with a suspension of *Methylobacterium* sp. 2A cells (0.05 OD_600nm_ units in 0.85% NaCl). Ten days later, the number of leaves and rosette diameter were determined and lateral root density was counted in each plant as the number of lateral roots/primary root length. For this, Petri dishes were photographed; the primary root length was measured and lateral roots were counted using Fiji software (Schindelin et al., 2012). Samples were stored in liquid nitrogen for further studies. Protein extracts were obtained and catalase activity was determined as described in Aebi (1984). Hydrogen peroxide (H_2_O_2_) quantification was performed according to Junglee et al. (2014). Protein content was estimated by Bradford using bovine serum albumin as standard. The experiment was conducted three times using two plates with five plants each for each condition (control, C; *Methylobacterium* sp. 2A-inoculated, I; salt stress, S; *Methylobacterium* sp. 2A-inoculated plants with salt stress, I+S).

### Plant Inoculation With *Methylobacterium* sp. 2A and Infection With *P. infestans*

*In vitro* four-weeks-old potato plants inoculated or not with *Methylobacterium* sp. 2A, were infected with *P. infestans* isolate Pi-60; 10-µl droplets of zoospore suspension were pipetted on three leaves per plant. Two flasks containing five plants each were used for each condition (Pi-60 and I+Pi-60). Five days later, *P. infestans* aggressiveness was observed (**Figure S2**). The result obtained encouraged us to conduct experiments in greenhouse plants. To this end, three-weeks-old potato plants were sprayed with *Methylobacterium* sp. 2A bacterial suspension (0.05 OD_600nm_ units in 0.85% NaCl), or with saline solution (control). Infection with Pi-60 was performed two days later; 10-µl droplets of sporangia suspension or water were pipetted on the abaxial side of apical leaflets of three leaves per plant (two equidistant spots per leaf). Five days post-infection (dpi), leaves were observed and photographed with a phone camera. Necrotic lesions were counted and Fiji software was used to measure the lesion area. The experiment was conducted twice using three plants for each condition (control, C; *Methylobacterium* sp. 2A-inoculated, I; infected, Pi-60; *Methylobacterium* sp. 2A-inoculated and infected, I+Pi-60).

Leaf samples were collected and total RNA was extracted using the TRIZOL Reagent (Invitrogen) following the manufacturer's instructions. Total RNAs (1 µg) were pre-treated with DNAse (RQ1 RNAse-free DNAse, Promega) and reverse transcribed with M-MLV-Reverse Transcriptase (Promega) using an oligo-dT primer and random hexamers. Expression levels of *StPR-1b* and *StPAL* genes were analyzed by RT-qPCR on an Applied Biosystems 7500 Real-Time PCR System, with the indicated primers (**Table S1**) (Genbiotech, Buenos Aires, Argentina) and FastStart Universal SYBR Green Master Rox (Roche). Elongation factor 1 alpha (*EF-1α*) was used as a reference gene. PCR reactions were incubated at 95˚C for 10 min followed by 40 cycles of 95˚C for 10s; and 60˚C for 1 min. PCR specificity was checked by melting curve analysis. Expression data was analyzed using the 2^–ΔΔCt^ method (Livak and Schmittgen, 2001).

### In Plate Confrontation Assay

Dual culture assays were performed in PDA or in RSA. *Methylobacterium* sp. 2A was striked on one half of the plate and, after 3 days of incubation at 25°C, a 1 cm^2^ plug from an actively growing culture of the oomycete *P. infestans* or the fungi *F. graminearum, B. cinerea, P. dissotocum,* and *R. solani,* was placed at the other half of the plate. Petri dishes with PDA or RSA containing only the corresponding plugs served as controls. The radial mycelial growth of the pathogens toward *Methylobacterium* sp. 2A (Ri) and that on a control plate (Rc) were measured and mycelial growth inhibition was calculated according to the formula: (Rc-R)/Rc x 100 (Lahlali and Hijri, 2010).

### *De Novo* Genome Assembly and Annotation

Whole Genome Sequencing (WGS) of *Methylobacterium* sp. 2A was carried out by an Illumina TruSeq Nano platform at Macrogen Laboratories. *De novo* assembly was done using the standard procedures from our own prokaryotic assembly pipeline (Sosa et al., 2018), based on SPAdes version 3.9.0 (Bankevich et al., 2012) and SSPACE version 3.0 (Boetzer et al., 2011). Genome annotation was done using the Rapid Annotations Subsystems Technology (RAST) server (Aziz et al., 2008).

### Whole-Genome Computational Analysis

The ANI (Average Nucleotide Identity) values between *Methylobacterium* sp. 2A and the other reference strains were calculated with the JspeciesWS web service (Richter et al., 2015). *In silico* DNA-DNA hybridization (DDH) was conducted between *Methylobacterium* sp. 2A and the reference strains with the Genome-to-Genome Distance Calculator web service (GGDC 2.1; Meier-Kolthoff et al., 2013).

### Search for Metabolic Pathways Associated With PGP Traits

On the basis of the annotated genome, computational prediction of metabolic pathways was made through automatic reconstruction by Pathway Tools v23.0. The Pathologic software of Pathway Tools (Karp et al., 2002) and the MetaCyc database (Caspi, 2005) were used to automatically generate a pathway-genome database (PGDB) from the GenBank file of the *Methylobacterium* sp. 2A annotated genome. The PGDB links the coding sequences and potential genes to enzymatic reactions and biochemical pathways. In addition, gene clusters were identified with SnapGene^4^ software (GSL Biotech) and with antiSMASH 5.0 (Blin et al., 2019) and manually curated.

### Assays for Detection of PGP Abilities

Dinitrogen fixation, phosphate solubilization, and IAA production were analyzed. The amount of IAA produced by *Methylobacterium* sp. 2A was estimated using Salkowski's method (Ehmann, 1977). The IAA-producing strains *Azospirillum brasilense* Az39 and *Pantoea* sp. were used as positive controls (Díaz Herrera et al., 2016). The different strains were grown in LB broth plus Trp (0.1 mg ml^-1^) and incubated at 28°C for 3, 4, or 5 days. After incubation, 2-ml aliquots were centrifuged and 1-ml supernatant samples were mixed with 1 ml of Salkowski's reagent (2% 0.5 FeCl_3_ in 35% H_2_SO_4_ solution) and kept in the dark. OD was recorded at 530 nm after 30 min. Nitrogen fixation was qualitatively determined by culturing single colonies in semisolid Nfb media, as described by Hartmann and Baldani (2006). An assay to evaluate phosphate solubilization was performed using tricalcium phosphate (TCP) in NBRIP solid and liquid media. Phosphate determination was made by the vanado-molybdate colorimetric method according to Pearson (1976). The strain *Pseudomonas fluorescens* BNM 233 currently used as a biofertilizer (Okon et al., 2015) was used as a positive control for phosphate solubilization.

### Statistical Analysis

Statistical analysis was performed by one-way or two-way ANOVA followed by Tukey's HSD test (p < 0.05) or by T-test as indicated in the figures, using GraphPad Prism^5^ version 5.03 (GraphPad Software, La Jolla, California, USA).

## Results

### General Features of *Methylobacterium* sp. 2A

**Figure 1:**
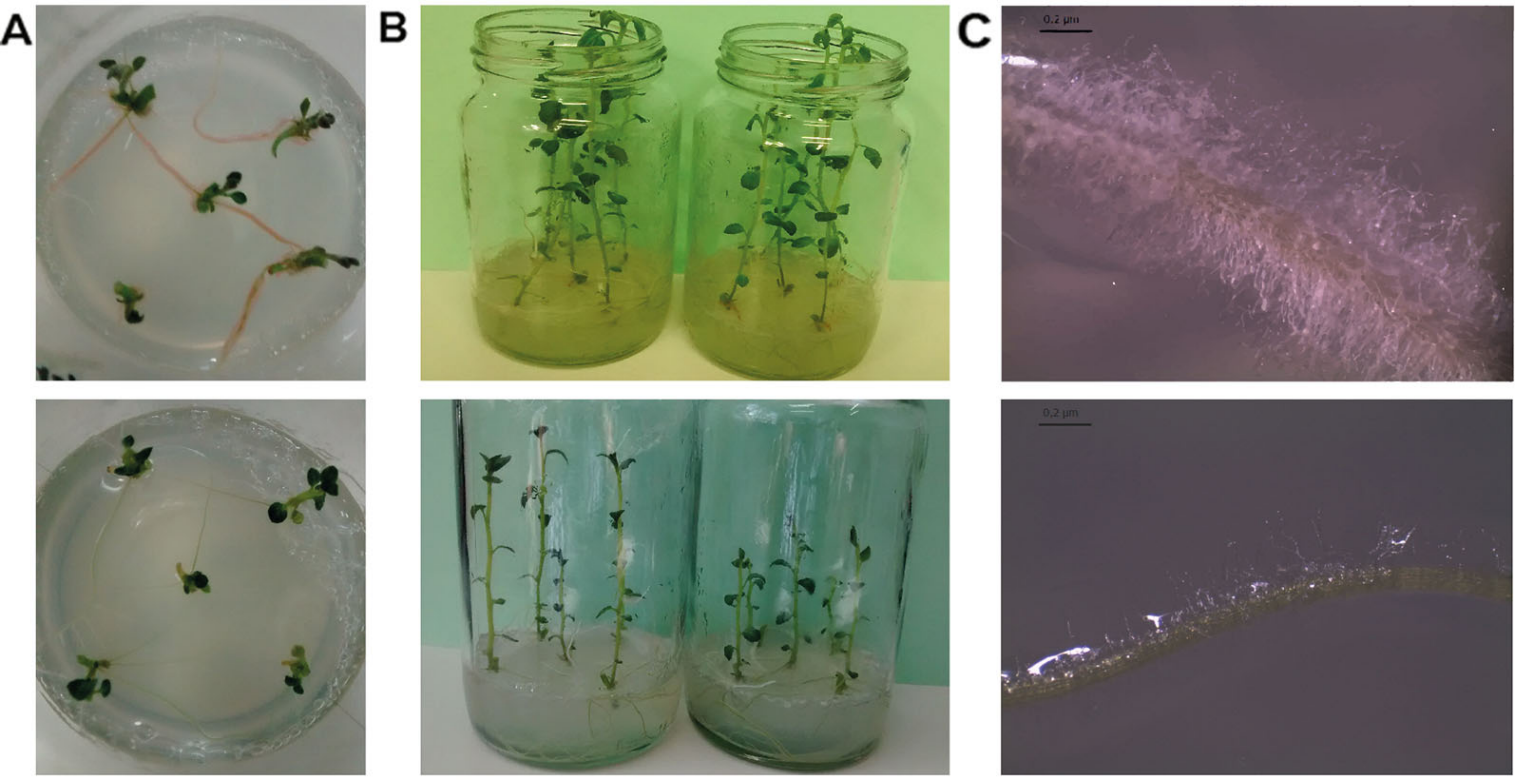
*Methylobacterium* sp. 2A was found associated with roots of potato plants established *in vitro.* **(A)** Bacterial pink-pigmentation was evident in the roots of one-week-old potato plants. Five-weeks-old plants associated with *Methylobacterium* sp. 2A exhibited increased growth **(B)** and increased root hair density **(C)**. Plants not associated with *Methylobacterium* sp. 2A that were micropropagated simultaneously are shown in lower panels as reference. Bar: 0.2 µm.

One-week-old potato plants micropropagated *in vitro* attracted our attention due to the pink-pigmentation of its roots (**Figure 1A**). MS media was not contaminated but a strong association with a microorganism was evident. One month later, these plants were more developed than other potato lines of the same age (**Figure 1B**) and the density of root hairs was increased (**Figure 1C**). A gram-negative bacillus, named *Methylobacterium* sp. 2A, was isolated from the roots and characterized. Colonies were pink-pigmented and circular, reaching a diameter of 0.2 mm after 3 days of incubation, denoting slow growth. It had a better performance in solid media containing plant extracts (PDA, PSA, and RSA media) while growth on LB agar was lower. Optimal growth was observed at 28°C, and the optimal pH growth range was between 5 and 7, being 6 the optimal pH value (**Table S2**). Oxidase and catalase reactions were positive. Resistance to hygromycin (10 µg/µl), ampicillin and chloramphenicol (20 µg/µl) was evidenced; however, *Methylobacterium* sp. 2A was sensitive to the other antibiotics tested.

### Phylogenetic Analysis of *Methylobacterium* sp. 2A

**Figure 2:**
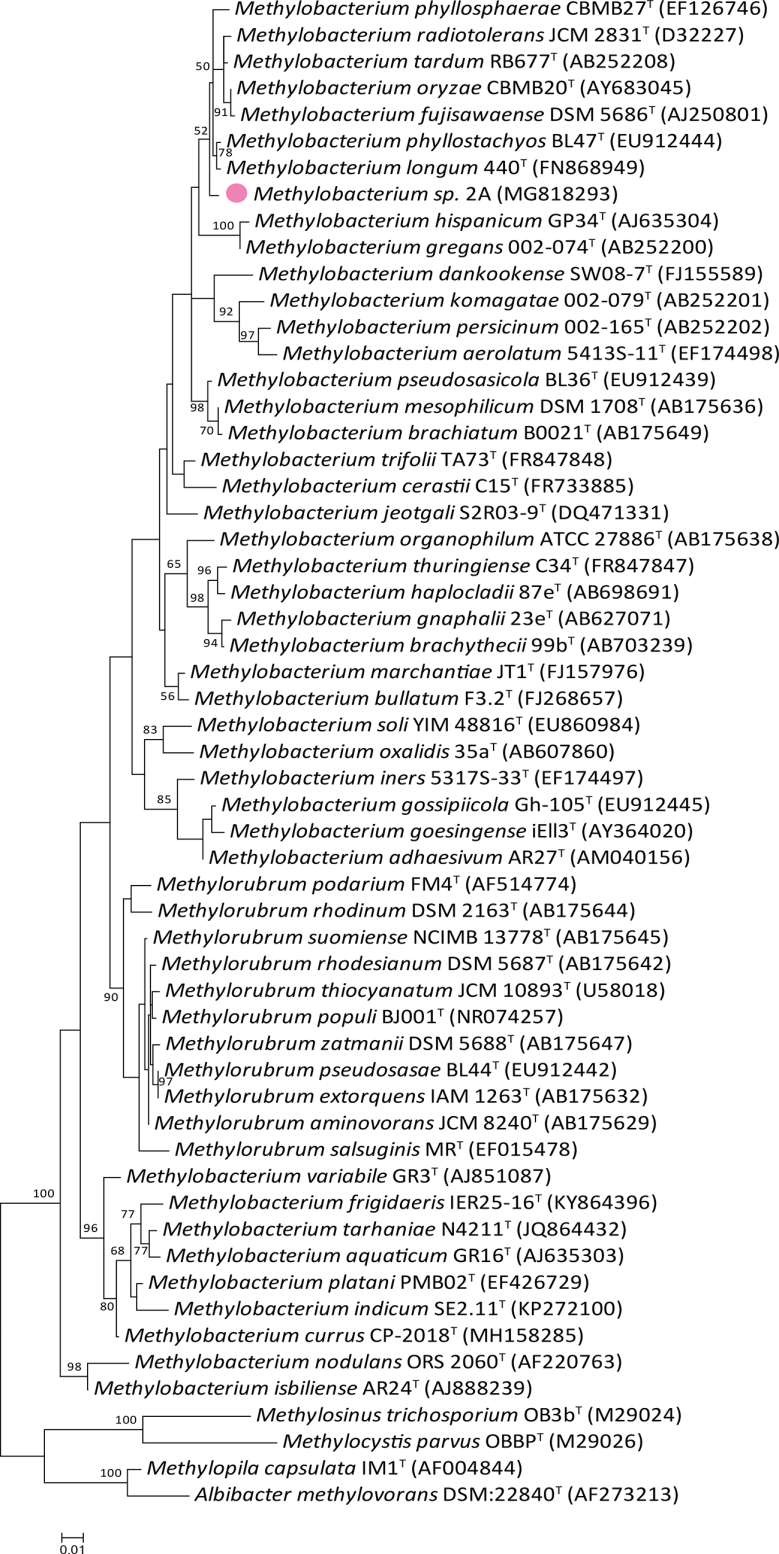
Phylogenetic tree of *Methylobacterium* sp. 2A and related members of the *Methylobacterium* genus based on *16S rDNA* gene sequence comparison. The maximum likelihood tree was reconstructed using the Kimura two-parameter model. The frequencies with which a given branch appeared in 1,000 bootstrap replications are indicated at nodes. Values below 50% are not displayed. Accession numbers are indicated in brackets. *Methylobacterium* sp. 2A position is indicated with a pink colored filled dot. Eleven species recently housed in the new *Methylorubrum* genus are included and four members of the Methylocystaceae family were used as outgroups.

The nearly full-length *16S rDNA* sequence (1,218 nt; GenBank accession number: MG818293.1) used for comparative gene sequence analysis, indicated that strain 2A belongs to the genus *Methylobacterium,* family Methylobacteriaceae, order Rhizobiales. EzBioCloud comparison revealed that *16S rDNA* from *Methylobacterium* sp. 2A shared high sequence similarity (>97%) with fourteen validated type species of the genus (**Table S3**). The phylogenetic tree (**Figure 2**) indicated that this isolate is most closely related to *M. fujisawaense*, *M. phyllosphaerae*, *M. oryzae, M. radiotolerans, M. tardum, M. longum,* and *M. phyllostachyos* (sequence similarity between 98.52% and 99.10%). Characteristics of *Methylobacterium* sp. 2A, such as colony pigmentation, cell size, growth conditions, and catalase and oxidase reactions, were compared (**Table S2**) with those of the most closely related species (Ito and Iizuka, 1971; Kato et al., 2008; Knief et al., 2012; Madhaiyan and Poonguzhali, 2014; Madhaiyan et al., 2007; Madhaiyan et al., 2009). As observed, though isolated from different sources, all strains were positive for catalase and oxidase reactions, had a similar pigmentation, and shared several growth conditions.

### Inoculation of *Methylobacterium* sp. 2A Conferred Stress Tolerance Under Salt Conditions

**Figure 3:**
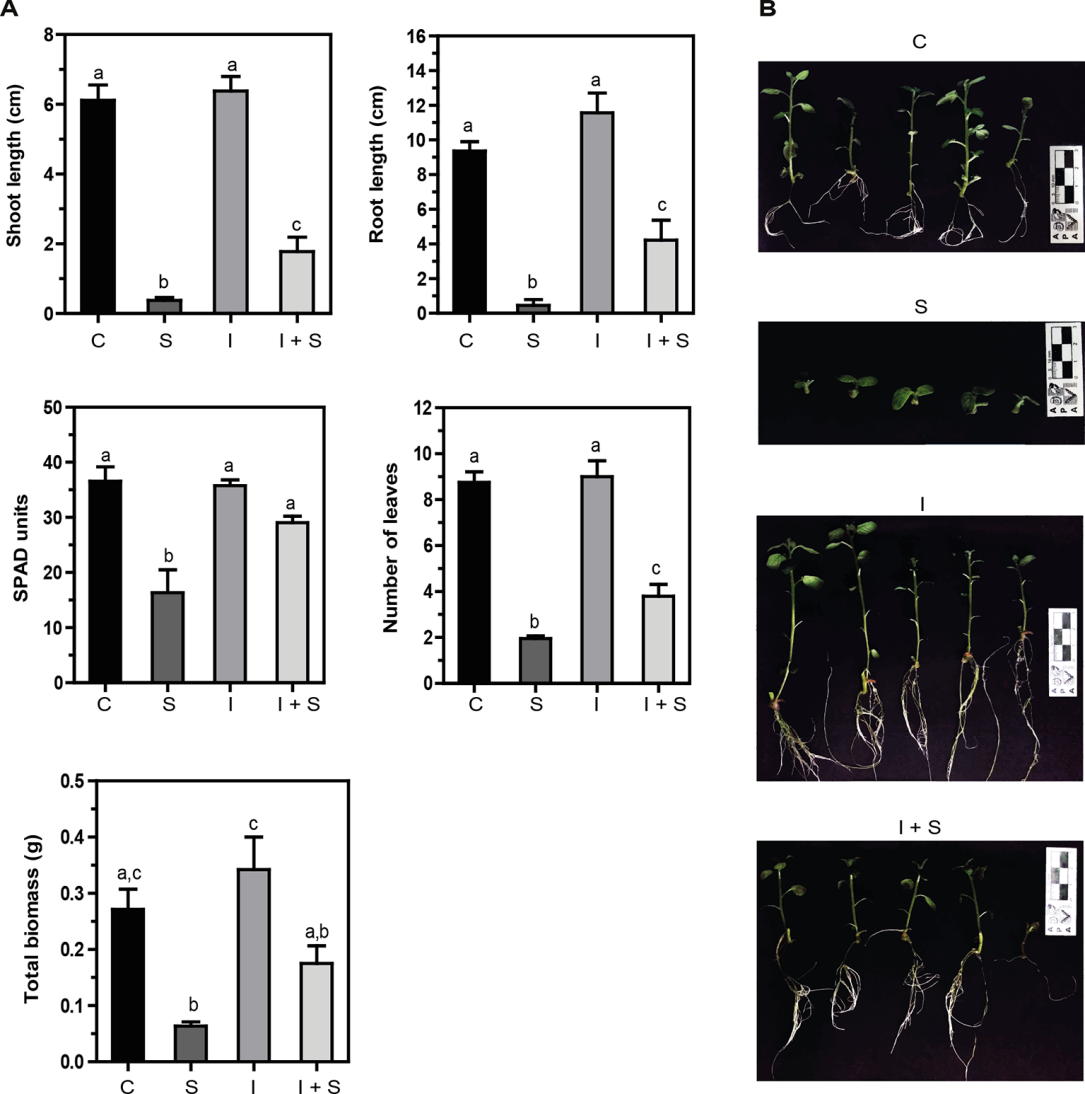
*Methylobacterium* sp. 2A mitigates salt stress in inoculated potato plants. Internodes from *in vitro* plants, inoculated or not with *Methylobacterium* sp. 2A, were grown in solid MS media (C or I) or in MS media with the addition of 50 mM NaCl (S or I+S). **(A)** Shoot and root length (cm), SPAD units, the number of leaves, and total biomass (g) were determined. Mean ± SEM of three biological replicates, with three technical replicates each, were plotted. Two-way ANOVA analysis was performed and Tukey´s HSD test was applied. Different letters above the bars indicate significant differences (p < 0.05). **(B)** Representative pictures of each condition are shown.

Salinity impairs plant growth and development *via* water stress, cytotoxicity due to excessive uptake of ions (Na^+^ and Cl^−^), and nutritional imbalance; it is accompanied by oxidative stress due to the generation of reactive oxygen species (ROS) (reviewed in Isayenkov and Maathuis, 2019). *In vitro* potato plants were grown in MS media with or without 50 mM NaCl for 21 days. Strong inhibition of shoot and root growth, and total biomass, together with a significant decrease in chlorophyll content (SPAD units) and leaf number (**Figures 3A, B**) was observed compared to control conditions (C vs. S). On the other hand, when the *Methylobacterium* sp. 2A-inoculated potato plants were grown in control (I) or salt stress conditions (I+S), the inhibition observed in shoot and root length and the decrease in the number of leaves and total biomass was less severe (I vs. I+S). Moreover, chlorophyll content was not reduced. When comparing noninoculated versus *Methylobacterium* sp. 2A-inoculated plants under salt conditions (S vs. I+S), a significant difference was observed in most parameters, indicating that this isolate is able to mitigate the negative effect of salinity.

**Figure 4:**
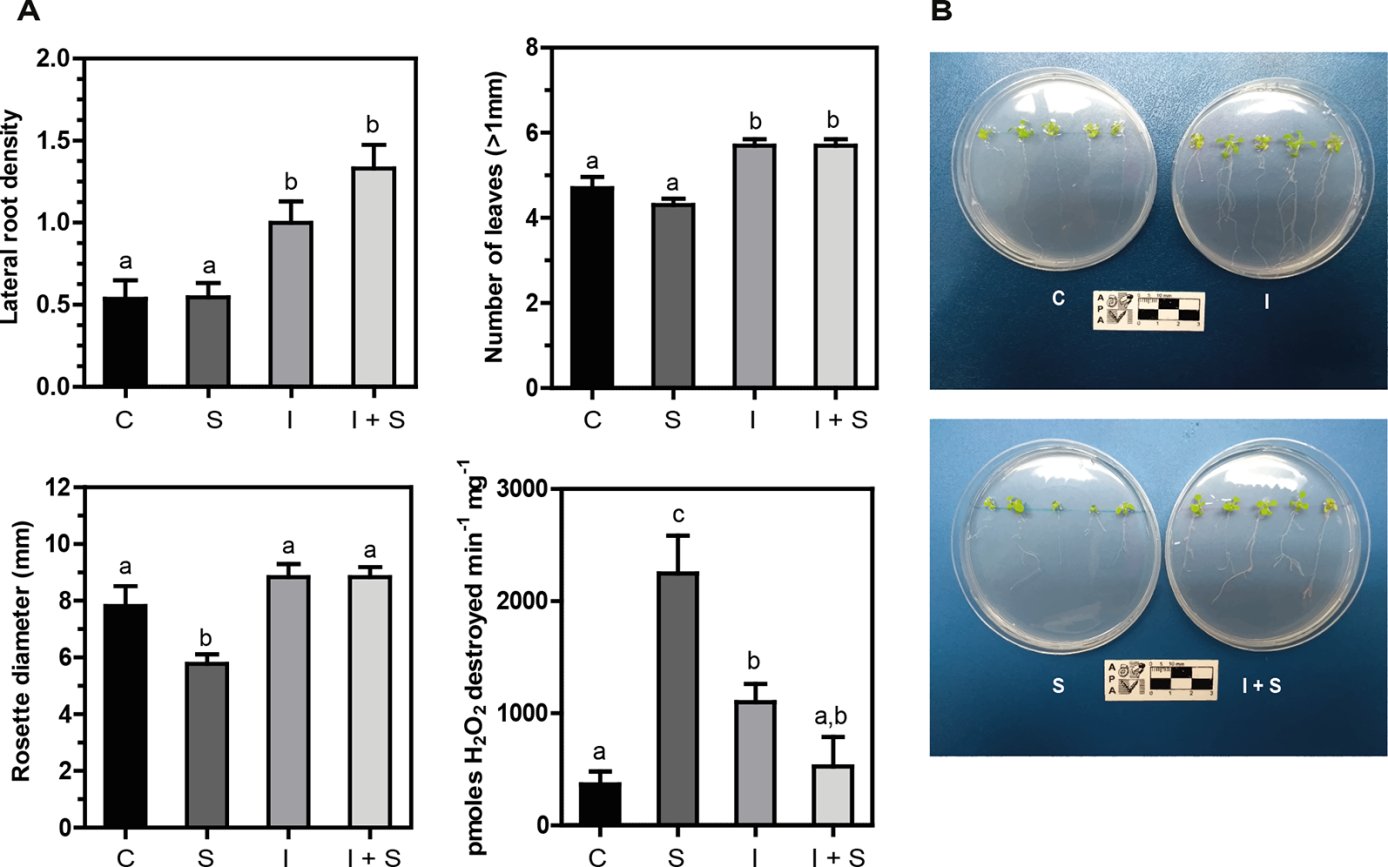
*Methylobacterium* sp. 2A mitigates salt stress in inoculated Arabidopsis plants. Col-0 seeds were inoculated or not with *Methylobacterium* sp. 2A and grown in solid MS media (C or I) or in MS media with the addition of 75 mM NaCl (S or I+S). **(A)** Lateral root density, the number of leaves, rosette diameter (mm), and catalase activity were determined. Catalase activity is expressed as pmoles H_2_O_2_ destroyed min^-1^ mg^-1^. Mean ± SEM of three biological replicates, with three technical replicates each, were plotted. Two-way ANOVA analysis was performed and Tukey´s HSD test was applied. Different letters above the bars indicate significant differences (p < 0.05). **(B)** Representative pictures of each condition are shown.

Arabidopsis plantlets inoculated or not with *Methylobacterium* sp. 2A were grown under control or salt stress conditions. After a week of conducting the experiment, a significant increase in lateral root density (p < 0.05) and in the number of leaves (p < 0.01) was observed in *Methylobacterium* sp. 2A-inoculated plants, both under control and stress conditions, compared to noninoculated ones (C vs. I, and S vs. I+S; **Figures 4A, B**). The reduction in rosette diameter observed in control plants under salt stress (C vs. S; **Figure 4A**) was not perceived when *Methylobacterium* sp. 2A was present (I vs. I+S). As depicted in **Figure 4A**, a sixfold increase in catalase activity was observed upon salt stress in noninoculated plants (C vs. S), but not in *Methylobacterium* sp. 2A-inoculated ones (C vs. I+S). However, upon inoculation, catalase activity increased three-fold under control conditions (C vs. I). The increase observed in catalase activity in stressed plants (S) was not sufficient to reduce H_2_O_2_ content, in fact, these plants present fivefold more peroxide than control ones (p = 0.01). However, no significant difference in peroxide content was observed when comparing control with *Methylobacterium* sp. 2A-inoculated plants under control (p = 0.656) or under salt stress conditions (p = 0.651). Furthermore, a similar peroxide content was observed in *Methylobacterium* sp. 2A-inoculated plants grown under control or saline conditions (p = 0.796). Our results indicate that this isolate can exert a salt-protective effect on different plant species.

### *Methylobacterium* sp. 2A Displays Biocontrol Activity Against *P. infestans*

**Figure 5:**
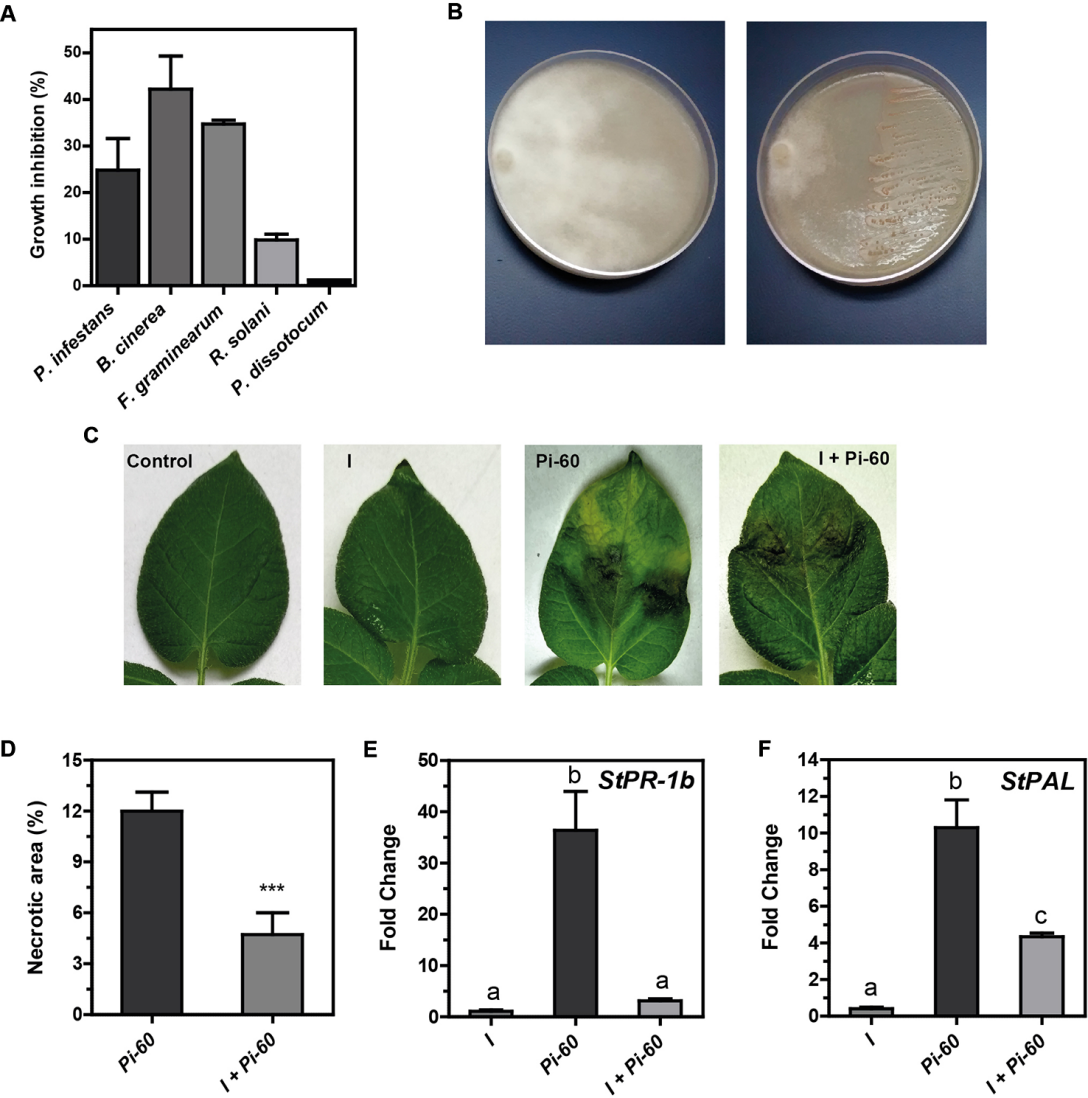
*Methylobacterium* sp. 2A is effective in controlling *P. infestans*. **(A)** Biocontrol activity of *Methylobacterium* sp. 2A against different phytopathogens in a dual confrontation assay. The mycelial growth inhibition (%) was calculated comparing the radial size of the pathogen colony in the dual culture and in control plates. **(B)** Illustrative image showing *Methylobacterium* sp. 2A antagonistic effect against *P. infestans*; left plate: pathogen; right plate: dual culture (pathogen + *Methylobacterium* sp. 2A). Images illustrating the antagonistic effect of *Methylobacterium* sp. 2A against the other pathogens are shown in **Figure S3**. **(C)** Greenhouse potato plants were inoculated or not with *Methylobacterium* sp. 2A 48 h prior to infection with *P. infestans*. Illustrative images show control, inoculated (I), infected (Pi-60) and inoculated and infected (I+Pi-60) apical leaflets. **(D)** Histogram depicting the percentage of necrotic area (%) in leaves infected with *P. infestans* that were previously inoculated (I+Pi-60) or not (Pi-60) with *Methylobacterium* sp. 2A. RT-qPCR analysis of *StPR-1b* **(E)** and *StPAL* **(F)** in inoculated leaves (I), in leaves infected with *P. infestans* (Pi-60) and in leaves inoculated with *Methylobacterium* sp. 2A and infected with *P. infestans* (I+Pi-60). EF-1α was used as a reference gene. Mean ± SEM of three biological replicates, with three technical replicates each, were plotted. Two-way ANOVA analysis was performed and Tukey´s HSD test was applied. Different letters above the bars indicate significant differences in transcript levels between treatments (p < 0.001).

PGPR can promote plant growth indirectly by preventing the deleterious effects of plant pathogens. Our result with *in vitro* plants suggested that *Methylobacterium* sp. 2A protected potato plants against *P. infestans* (**Figure S2**). In order to evaluate the biocontrol effect of this strain, an in plate confrontation assay against *P. infestans, B. cinerea, Fusarium* sp.*, R. solani,* and *P. dissotocum* was performed. As shown in **Figures 5A, B** and **S3**, *Methylobacterium* sp. 2A inhibited mycelial growth of *P. infestans* (24.8%), *B. cinerea* (42.1%), and *Fusarium* sp. (34.7%). On the contrary, no notorious effect was observed against *R. solani* (7.2%) and *P. dissotocum* (1.2%).

To further analyze the antagonistic effect of *Methylobacterium* sp. 2A, *P.infestans* infection assays were conducted on greenhouse plants that were previously sprayed with *Methylobacterium* sp. 2A (I) or with water (controls, C). At 5 dpi we evaluated the presence and area of necrotic lesions (**Figures 5C, D**). No lesions were observed in leaves sprayed with *Methylobacterium* sp. 2A (I), while lesions were evident in *P. infestans* treated ones (Pi-60 and I+Pi-60). However, lesion size was significantly bigger in the absence of *Methylobacterium* sp. 2A and 87.5% of infected leaves presented chlorosis. On the other hand, in *Methylobacterium* sp. 2A-sprayed plants, only 28.5% of the leaves were chlorotic. This result suggests that *Methylobacterium* sp. 2A is able to restrict *P. infestans* growth.

Induced systemic resistance (ISR) is a physiological “state of enhanced defensive capacity” elicited by PGPR where the plant's innate defenses are potentiated against subsequent biotic challenges (reviewed in Pieterse et al., 2014). We previously reported that the pathogenesis-related protein 1b (*StPR-1b*) and phenylalanine ammonia-lyase (*StPAL*) genes were induced in infected and in distal potato leaves upon *P. infestans* infection (Fantino et al., 2017). Therefore, we decided to analyze their expression in leaves of plants sprayed with *Methylobacterium* sp. 2A that were then infected or not with Pi-60 and compare them with leaves infected with Pi-60 that were not previously inoculated (**Figures 5E, F**). At the time point analyzed, no induction of *StPR-1b* or *StPAL* expression was observed in inoculated leaves (I). *StPR-1b* expression was strongly upregulated (*ca.* 35-fold, *p* < 0.001) in the presence of the oomycete (Pi-60), however, no upregulation was observed when *P. infestans* zoospores were spotted in leaves previously sprayed with *Methylobacterium* sp. 2A (I+Pi-60). On the other hand, *StPAL* was induced tenfold in infected leaves (Pi-60), and fourfold in I+Pi-60 leaves. The reduction observed in the expression of *PR-1b* upon *P. infestans* infection in *Methylobacterium* sp. 2A-inoculated plants suggests that the defense mechanisms triggered by this isolate do not involve this gene.

### General Genome Features of *Methylobacterium* sp. 2A

Our data indicated that this isolate has the potential to be a PGPR, so we decided to sequence its genome in order to identify the genes that could be responsible for this effect. *Methylobacterium* sp. 2A genomic sequence is 6,395,352 bp in length, and G+C content is 69.34 mol%. The ANI values between *Methylobacterium* sp. 2A and *M. phyllosphaerae* CBMB27 (GenBank accession no. NZ_CP015367.1), *M. oryzae* CBMB20 (GenBank accession no. NZ_CP003811.1*), M. radiotolerans* JCM 2831 (GenBank accession no. NC_010505.1), and *M. phyllostachyos* BL47 (GenBank accession no. NZ_FNHS00000000.1) were 84.53%, 84.37%, 85.21%, and 87.79%, respectively. These results were lower than the established threshold of 95%–96% ANI for prokaryotic species boundary (Chun et al., 2018). Furthermore, *in silico* DDH values between *M. phyllosphaerae, M. oryzae, M. radiotolerans,* and *M. phyllostachyos* were 30.50, 30.50, 31.00, and 37.00, respectively. The abovementioned genomic results suggest that this species has not been described so far.

A total of 6,142 coding DNA sequences (CDSs) and 55 structural RNAs (50 tRNAs) were predicted; 2,022 (33%) were classified as hypothetical and 2,419 CDSs (40%) were assigned to RAST subsystems (**Figure S4**). Using the GenBank file of the annotated genome as input, the Pathologic software automatically assigned 306 pathways and 2,116 enzymatic reactions.

### Plant Growth Promotion Traits in *Methylobacterium* sp. 2A Genome

Genes involved in chemotaxis and motility were found in *Methylobacterium* sp. 2A genome; there are 52 genes encoding methyl-accepting chemotaxis proteins (MCP), a *cheV*, the *cheAWRB* gene cluster, and *cheY*. The chemotaxis response regulator *cheA* gene is essential for motility toward root exudates (De Weert et al., 2002). Moreover, it has 31 flagellar-related genes grouped in at least five clusters (**Table S4**). As other methylotrophic bacteria, *Methylobacterium* sp. 2A genome contains the *mxa* cluster (12,475 bp) responsible for methanol oxidation.

**Figure 6:**
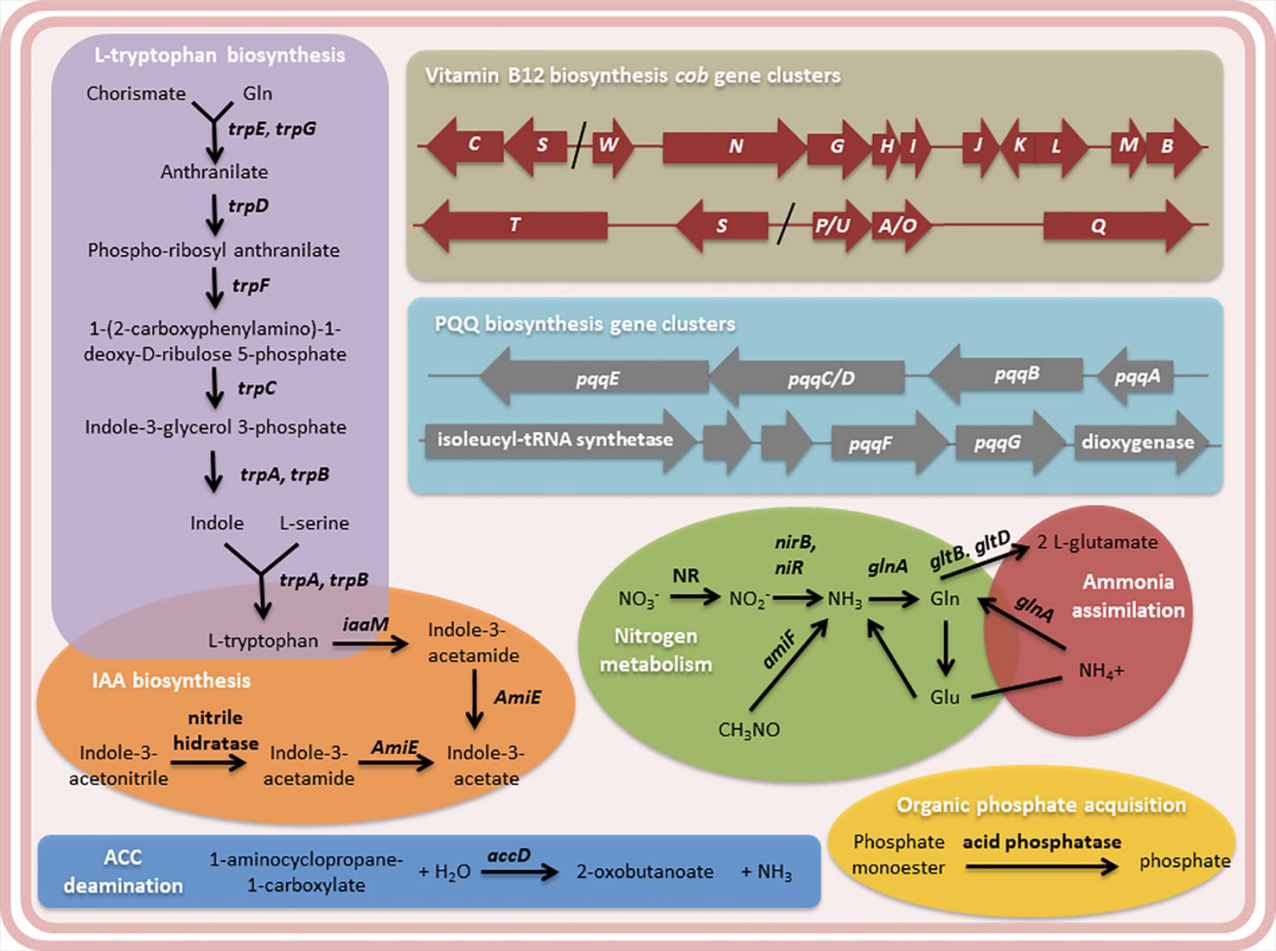
The genome of *Methylobacterium* sp. 2A contains genes that may contribute to plant growth stimulation and fertilization. Metabolic pathways related to L-tryptophan and indole acetic acid (IAA) biosynthesis, and N_2_ metabolism and ammonia assimilation pathways are shown. The *cob* gene clusters involved in vitamin B12 biosynthesis and the pyrroloquinoline quinone (*PQQ*) operons are indicated. PQQ is a cofactor of GDH, involved in phosphate solubilization. 1-aminocyclopropane-1-carboxylate (ACC)-deamination and organic phosphate acquisition reactions are included. Gene annotation, EC number, and KEGG gene names of the enzymes mentioned in the diagram are listed in **Table S4**.

In addition, several genes were found in the *Methylobacterium* sp. 2A genome attributable to the PGP traits observed in potato and Arabidopsis plants (**Figure 6** and **Table S4**). We identified the five enzymes involved in L-tryptophan biosynthesis that are encoded by seven genes (*trp A-G)* in this isolate as in all microbial genomes. Though the chemical reaction steps are highly conserved, the genes of the pathway enzymes show considerable variations in arrangements, operon structure, and regulation in diverse microbial genomes (Priya et al., 2014). In *Methylobacterium* sp. 2A, *TrpEGDC* genes are clustered in an operon while *trp A, B,* and *F* are unconnected. L-tryptophan is the auxin (IAA) precursor.

**Figure 7:**
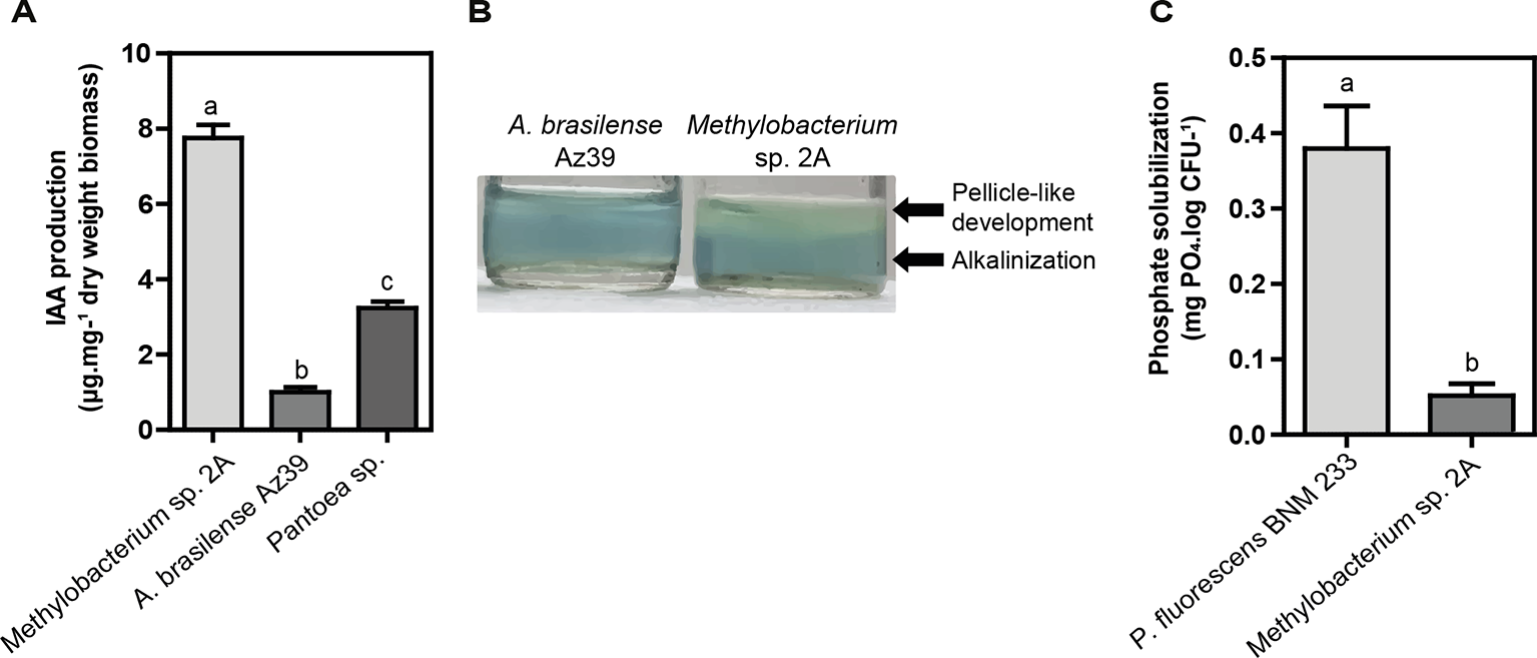
Plant growth-promoting (PGP) abilities of *Methylobacterium* sp. 2A. **(A)** *Methylobacterium* sp. 2A produced higher indole acetic acid (IAA) amounts than *A. brasilense* Az39 and *Pantoea* sp. strains used as positive controls. **(B)** The nitrogen-fixing ability of *Methylobacterium* sp. 2A was inferred from its growth in a nitrogen-free culture medium (Nfb); the pH change observed was similar to that of the well-known nitrogen-fixing bacterium *A. brasilense*. **(C)** Inorganic phosphate solubilization was confirmed in liquid NBRIP medium. *P. fluorescens* strain BNM 233 was used as positive control. One-way ANOVA analysis was performed and Tukey´s HSD test was applied. Different letters above the bars indicate significant differences (p < 0.05).

Two proposed IAA biosynthesis pathways were identified: the indole-3-acetamide (IAM) and the indole-3-acetonitrile (IAN) pathways. The IAM pathway consists of two-steps were L-tryptophan is first converted to IAM by the enzyme tryptophan-2-monooxygenase *(IaaM),* and then IAM is converted to IAA by an aliphatic amidase *(AmiE)*. In the IAN pathway, IAN can first be converted to indole-3-acetamide (IAM) by nitrile hydratase and then IAM is converted to IAA by an aliphatic amidase. Also, a nitrilase enzyme has been suggested to convert IAN to IAA directly without the IAM intermediate step. The production of IAA was confirmed using the Salkowski reagent. In fact, *Methylobacterium* sp. 2A produced higher levels of IAA than the *A. brasilense* Az39 and *Pantoea* sp. strains (**Figure 7A**). This tightly correlates with the increase observed in root hair development in potato and in lateral root density in Arabidopsis. As many PGPR associated with plant roots, *Methylobacterium* sp. 2A contains the gene encoding 1-aminocyclopropane-1-carboxylate (ACC) deaminase that can metabolize ACC, the immediate precursor of ethylene, thereby decreasing plant ethylene production and increasing plant growth.

Cobalamin (Vitamin B_12_) has been suggested to stimulate plant development and could be synthesized either *via de novo* or salvage pathways. For *de novo* biosynthesis more than thirty enzymatic steps are required, while the salvage pathway is a cost-effective way for bacteria (Palacios et al., 2014). In the *Methylobacterium* sp. 2A genome, all the genes involved in the aerobic *de novo* biosynthesis were identified (**Table S4**); some of them are grouped in clusters of genes: *cobP/UA/OQ*, *cobST, cobSC,* and *cobWNGHIJKLMB* (**Figure 6**). *Methylobacterium* sp. 2A harbors two copies of *cobS*. In gram-negative bacteria, exogenous corrinoids are transported into the cell through an ATP-binding cassette (ABC) transport system consisting of *Btu* genes. *BtuB*, *BtuC,* and *BtuN* genes coding for receptors were identified suggesting that this strain can also synthesize cobalamin by absorbing corrinoids *via* the salvage pathway (**Table S4**).

On the other hand, *Methylobacterium* sp. 2A presents the two-component regulatory genes involved in nitrogen fixation, *NtrBC*, and *NtrXY* (**Table S4**). This system allows the survival of the bacteria in a nitrogen-depleted medium. In fact, we confirmed that *Methylobacterium* sp. 2A is able to grow in NFb medium (**Figure 7B**). A two-component nitrogen fixation transcriptional regulator, *FixJ* and a *FixL* gene were identified in its genome, together with *nifJ*, *nifP*, *nifS,* and *nifU* genes (**Table S4**); however, other *nif* genes were not assigned by the RAST annotation. In addition, it harbors the enzymes involved in nitrogen metabolism and ammonia assimilation (**Figure 6**, **Table S4**).

Moreover, there are four glucose dehydrogenase (*gdh*) genes present in *Methylobacterium* sp. 2A genome (**Table S4**). GDH requires pyrroloquinoline-quinone (PQQ) as a redox cofactor for direct oxidation of glucose to gluconic acid, which diffuses and helps in acidic solubilization of mineral phosphates in soil. Synthesis of PQQ requires the expression of six genes, *pqqA*-*G* (Puehringer et al., 2008). *Methylobacterium* sp. 2A harbors the complete *pqqABC/DE* operon where *pqqC* and *pqqD* are fused into a single polypeptide designated *pqqC/D* (**Figure 6**, **Table S4**) as in *Methylorubrum extorquens* AM1 (Toyama et al., 1997). In *M. extorquens* the cluster containing *pqqF/G* is a group of six genes transcribed in the same direction, the first is predicted to encode an isoleucyl tRNA synthetase and the sixth a dioxygenase (Zhang and Lidstrom, 2003). In *Methylobacterium* sp. 2A we identified this same region (**Figure 6**). The DNA sequence encoding the last five genes shared 84.65% identity with the *M. extorquens* cluster. In addition, there are three kinds of microbial enzymes that can solubilize organic phosphate: a nonspecific acid phosphatase, a phytase, and C-P lyase or phosphonatase. *Methylobacterium* sp. 2A genome has three genes encoding acid phosphatases that can release phosphate from phosphoric ester or phosphoric anhydride (**Figure 6**).

Phosphate solubilization is an important trait to increase the availability and uptake of mineral nutrients for plants. From the genomic data, we can infer that *Methylobacterium* sp. 2A is able to solubilize phosphate, so we grew it in NBRIP solid and liquid media containing TCP. In the solid media, a yellow halo indicative of phosphate solubilization was observed around the positive control, *P. fluorescens* BNM 233 colony, but not around *Methylobacterium* sp. 2A. On the other hand, when grown in liquid media, *Methylobacterium* sp. 2A was able to solubilize mineral phosphate but to a lower extent than the positive control (**Figure 7C**).

In addition, antiSMASH analysis identified 10 genes associated with terpene biosynthesis (four phytoene synthases, four phytoene desaturases, one phytoene cyclase, and one phytoene dehydrogenase) in three genomic regions. Recent reports highlight the ecological importance of microbial terpenes emitted by beneficial microorganisms that function as volatile organic compounds (mVOCs) altering plant development (reviewed in Schulz-Bohm et al., 2017).

The dual confrontation assays and the *in planta* infection assays suggest that this isolate may produce compounds to reduce the growth of phytopathogens. In fact, its genome carries a chitinase, a phenazine biosynthesis *PhzF* gene, two genes involved in 4-hydroxybenzoate synthesis (the transferase, *ubiA*, and a transporter), a penicillin acylase (PAH), and a *CvpA* gene encoding a Colicin V production protein. Several copies of the genes responsible for translocating colicins across the membrane (*TolA*, *TolB, TolC, TonB*) were also present (**Table S4**). In addition, genes responsible for aerobactin and enterobactin biosynthesis are present in its genome (**Table S4**). The presence of a siderophore biosynthetic gene cluster (*IucABCD*) and a siderophore transporter protein was confirmed with antiSMASH analysis. Concerning antibiotic resistance, *Methylobacterium* sp. 2A has two chloramphenicol acetyltransferases consistent with the observed chloramphenicol resistance, and a B-lactamase gene that could be responsible for the ampicillin resistance.

Furthermore, its genome encodes ROS scavenging enzymes that could alleviate oxidative damage: eleven catalases, two glutathione reductases, two superoxide dismutases, and five peroxidases, among them, are two glutathione, a cytochrome c, and a thiol peroxidase (**Table S4**).

## Discussion

PGPR stimulate plant growth by either providing plant hormones (auxin or cytokinin), lowering plant ethylene levels through the action of the enzyme 1-aminocyclopropane-1-carboxylate (ACC) deaminase, helping in the acquisition of nutritional resources (nitrogen, iron, and phosphorus), or antagonizing phytopathogens (Glick, 1995; Glick et al., 1999). *In vitro* potato and Arabidopsis plants inoculated with *Methylobacterium* sp. 2A were more developed than noninoculated plants of the same age, suggesting that this isolate could stimulate plant growth. Genome sequencing indicates that *Methylobacterium* sp. 2A presents several traits related to phyto-stimulation and phyto-fertilization, such as L-tryptophan and IAA biosynthesis, phosphate acquisition and solubilization, siderophore and vitamin B12 biosynthesis, N_2_ fixation and ammonia assimilation. In fact, the increase observed in root hair development and in lateral root density are auxin-associated phenotypes (Overvoorde et al., 2010) that are in agreement with the IAA produced by this isolate.

The experiments presented in this work were conducted using *in vitro* plants; however, preliminary experiments were performed with tubers inoculated with *Methylobacterium* sp. 2A planted in the greenhouse. These plants were more developed than noninoculated ones (data not shown). Apart from modifying IAA levels, this isolate could stimulate plant growth either by fixing N_2_ or by improving phosphate acquisition. We recognized several genes involved in fixing N_2_ in *Methylobacterium* sp. 2A; however, we could not identify the *NifH* gene of nitrogenase. Multiple independent horizontal gene transfer events took place in the evolution of nitrogen-fixing bacteria. Leaf isolates from the *Methylobacterium* genus present sequences highly divergent from *NifH* consensus sequence, more related to the Pfam NifH/frxC-family protein, such as BchX (Madhaiyan et al., 2015). *Methylobacterium* sp. 2A harbors a chlorophyllide reductase iron protein subunit X (BchX); this fact, together with its ability to grow in Nfb medium, suggests that it is a diazotrophic bacterium. Nevertheless, the nitrogenase activity of *Methylobacterium* sp. 2A should be assessed by the acetylene reduction assay to conclude this.

Potato is a high fertilizer-demanding crop, which requires 250 kg ha^−1^ of nitrogen and 150 kg ha^−1^ of phosphorus to get an optimum yield (Khan et al., 2012). Phosphorus availability is often limited due to the formation of insoluble inorganic and organic phosphate complexes (Adesemoye and Kloepper, 2009). The genomic data indicate that *Methylobacterium* sp. 2A is able to mobilize organic sources of phosphorus *via* acid phosphatases, and solubilize mineral phosphate, thus contributing to plant phosphate acquisition. The solubilization assay confirmed that this isolate is able to solubilize inorganic phosphate. Therefore, *Methylobacterium* sp. 2A emerges as a potential biofertilizer for potato plants to improve phosphorus and nitrogen uptake as reported for other PGPR (Hanif et al., 2015; Naqqash et al., 2016).

Furthermore, our results indicate that *Methylobacterium* sp. 2A mitigates the deleterious effects of salt stress in potato and Arabidopsis plants. Under stress conditions, ethylene regulates plant homeostasis and reduces root and shoot growth. However, under saline conditions, *Methylobacterium* sp. 2A-inoculated plants presented higher biomass, increased root and shoot growth, and more chlorophyll content than noninoculated plants. The modification in root-architecture promoted by auxin possibly increases the uptake of water and nutrients, thus explaining the improved fitness of the plants. In addition, like many other PGPRs, this isolate encodes an ACC deaminase; it was reported that degradation of ACC by bacterial ACC deaminase releases plant stress and rescues normal plant growth (Glick et al., 2007). In particular, the ACC deaminase-producing bacteria *Achromobacter piechaudii* ARV8, lowered the level of ethylene and prevented inhibition of plant growth when inoculated in tomato plants grown in the presence of high salt (Mayak et al., 2004a) and drought stress (Mayak et al., 2004b). Therefore *Methylobacterium* sp. 2A could alleviate salt stress by increasing auxins and decreasing ethylene content in the rhizosphere.

Another important feature observed was that when grown under salt stress, *Methylobacterium* sp. 2A-inoculated Arabidopsis plants presented lower levels of peroxide than noninoculated ones. Its genome contains several ROS scavenging enzymes and was positive for catalase reaction. It was reported that ROS degradation could be the result of enhancing plant antioxidant activities by PGPR thus protecting plants from salt toxicity (Chu et al., 2019). Catalase activity increased upon inoculation with *Methylobacterium* sp. 2A in control conditions, but its activity was lower than in noninoculated plants when exposed to salt stress. A meta-analysis performed by Pan et al. (2019) suggests that PGPR helps host plants to alleviate oxidative stress mainly through reducing the generation of ROS formed on the onset of ionic stress, not *via* scavenging ROS by accumulating antioxidant enzymes in host plants. This might be the case for *Methylobacterium* sp. 2A.

To successfully thrive within bacterial populations, *Methylobacterium* sp. 2A has to be highly competitive. Its genome harbors bacterial chemotaxis and motility genes, and several genes involved in the production of antimicrobial compounds (listed in **Table S4**). The first could be responsible for root association, and the later could function as antibiotics conferring an advantage against other microorganisms while protecting the plant against phytopathogens. In this regard, chitinase has been associated with protection against plant fungal pathogens (Kumar et al., 2018); colicins are the most representative bacteriocins produced by gram-negative bacteria (Beneduzi et al., 2012), while chemicals such as phenazine and 4-hydroxybenzoate act as antibiotics and suppress plant pathogenic microbes (Gupta et al., 2015). As an example, phenazine is known to suppress the plant pathogen *F. oxysporum* (Chin-A-Woeng et al., 2003). It was reported that PAH participates in acyl-homoserine lactones degradation thus interfering with quorum sensing of competing bacteria (Mukherji et al., 2014). Genes related to aerobactin and enterobactin biosynthesis were also identified in the *Methylobacterium* sp. 2A genome. Siderophore producing bacteria are of significant importance in the field of agriculture; in addition to supplementing iron to the plant, siderophores prevent the growth of the soil-borne phytopathogens (Fernández Scavino and Pedraza, 2013).

The biocontrol capacity of *Methylobacterium* sp. 2A was evidenced in the dual confrontation assays against *B. cinerea*, *F. graminearum* and *P. infestans*, and in greenhouse plants infected with *P. infestans*. Lesion size was smaller and chlorosis was seldom observed in *Methylobacterium* sp. 2A-inoculated plants infected with Pi-60 compared to noninoculated plants. In addition to producing antimicrobial compounds, PGPR are capable of potentiating plant defense mechanisms against pathogens. ISR has been reported in potato after inoculation with a Rhizobium strain (Reitz et al., 2001). This response differs from systemic acquired resistance (SAR) and is not salicylic acid (SA) dependent (Pieterse et al., 2014). In fact, at the time points analyzed we observed that *St-PR1b* was not induced in *Methylobacterium* sp. 2A-inoculated leaves after infection with Pi-60. On the contrary, a very strong induction of *StPR-1b* was observed when noninoculated plants were infected with *P. infestans*. This induction, however, did not restrain the oomycete growth. On the other hand, induction of *StPAL* was observed in *Methylobacterium* sp. 2A-inoculated plants upon infection with *P. infestans*. While its antagonizing effect is clear, at the moment, we cannot infer which plant defense mechanisms are primed by *Methylobacterium* sp. 2A.

In this work, we present a new PGPR isolate capable of promoting plant growth under control and salt stress conditions in two dicots. Probably this beneficial effect is not limited to potato and Arabidopsis plants. In addition, in dual confrontation assays, *Methylobacterium* sp. 2A restricted the growth of two necrotrophic fungi and of the hemibiotrophic oomycete *P. infestans*, denoting broad spectrum antagonism. *In planta* assays confirmed that it was able to reduce *P. infestans* deleterious effect in potato plants. These promissory results allow us to envisage that *Methylobacterium* sp. 2A has the potential to be used as a substitute for chemical fertilizers and fungicides, preventing pollution in farmlands. This research is the first approach to understand the PGP capacities of *Methylobacterium* sp. 2A; we have to test this isolate in the field in order to assess the productivity, efficacy, and viability of this inoculum. Certainly, this eco-friendly microbial technology will contribute to sustainable agricultural practices and is an alternative strategy to improve crop production for an increasing world population.

## Data Availability Statement

This whole-genome shotgun project has been deposited at DDBJ/EMBL/GenBank under the BioProject PRJNA560067. The version described in this paper is version VUOK00000000.1.

## Author Contributions

RU conceived and designed the research. CG, EF, and MZ performed and designed the experiments. CG, FS, and DF performed computational analyses. RU and CG analyzed the genomic data and wrote the manuscript. All authors have read and approved the final manuscript.

## Funding

RU and DF are members of Carrera de Investigador Científico from Consejo Nacional de Investigaciones Científicas y Técnicas (CONICET, Argentina); RU is Associate Professor at Universidad de Buenos Aires (UBA). MZ is Professor and Researcher at UBA. CG and FS are recipients of a doctoral scholarship from CONICET and EF is a postdoctoral fellow from CONICET. This work was funded by CONICET (PIP 0455), Universidad de Buenos Aires (UBACYT), and Agencia Nacional de Promoción Científica y Tecnológica (PICT-2014 3018).

## Conflict of Interest

The authors declare that the research was conducted in the absence of any commercial or financial relationships that could be construed as a potential conflict of interest.

## Footnotes

1
http://fao.org

2
http://ourworldindata.org

3
http://cipotato.org

4
http://snapgene.com

5
http://graphpad.com
