# Supplementary material for: Methylobacterium sp. 2A Is a Plant Growth-Promoting Rhizobacteria That Has the Potential to Improve Potato Crop Yield Under Adverse Conditions
Source: Front Plant Sci. 2020 Feb 14;11:71. doi: 10.3389/fpls.2020.00071 (PMC7038796; doi:10.3389/fpls.2020.00071)
Supplement: Supplementary file 1 [file DataSheet_1.docx]

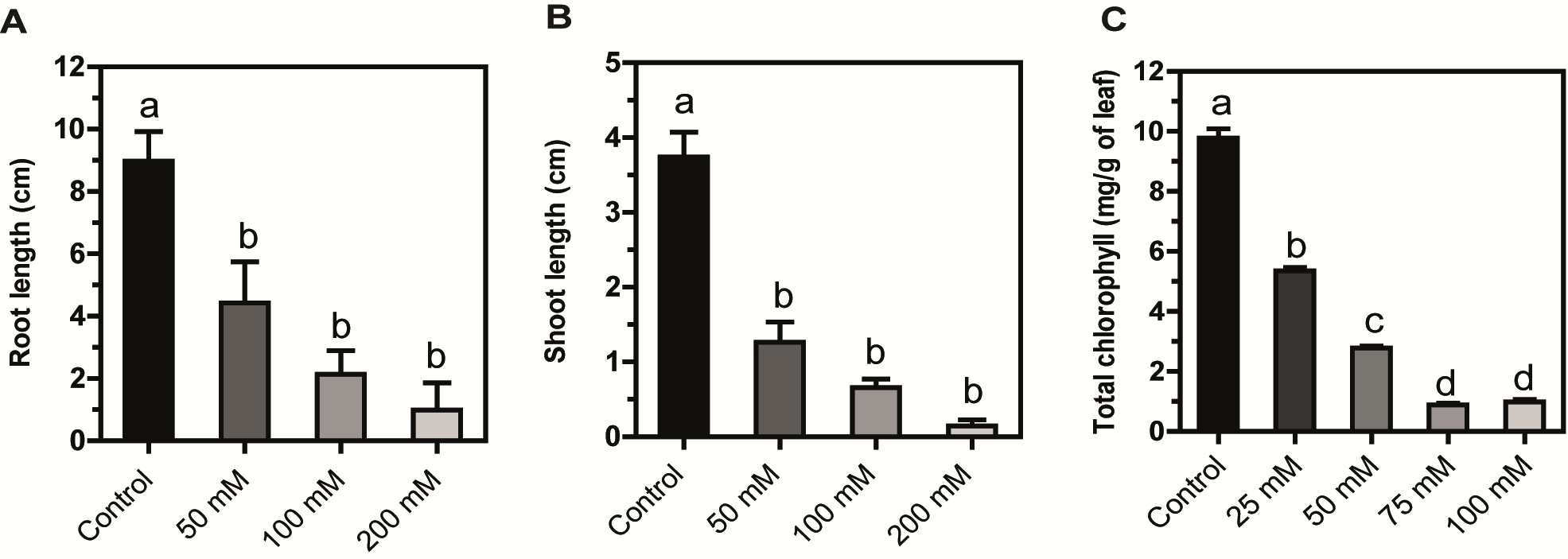


**Figure S1. Potato internodes were grown at increasing NaCl concentrations.** **(A)** Root length, **(B)** shoot length and, **(C)** total chlorophyll were measured in order to find a salt concentration where stress was evidenced. Different letters above the bars indicate significant differences.
