# Supplementary material for: Methylobacterium sp. 2A Is a Plant Growth-Promoting Rhizobacteria That Has the Potential to Improve Potato Crop Yield Under Adverse Conditions
Source: Front Plant Sci. 2020 Feb 14;11:71. doi: 10.3389/fpls.2020.00071 (PMC7038796; doi:10.3389/fpls.2020.00071)
Supplement: Supplementary file 2 [file DataSheet_2.docx]

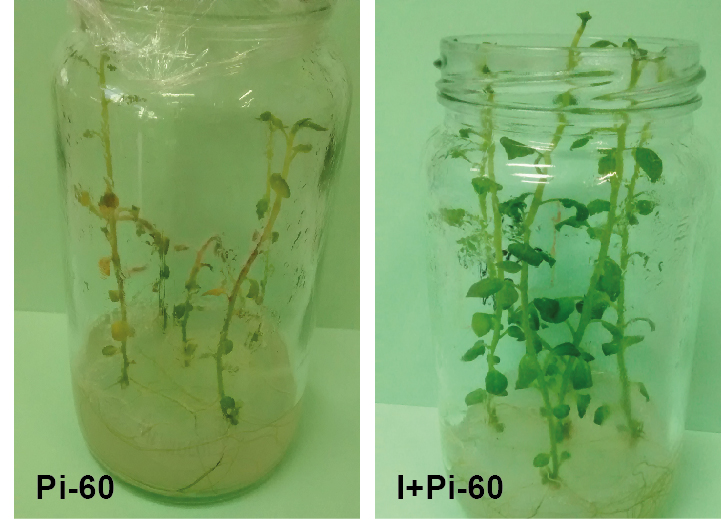


**Figure S2. *Methylobacterium* sp. 2A protected potato plants against *P. infestans.*** *In vitro* four-weeks-old potato plants inoculated or not with *Methylobacterium* sp. 2A, were infected with *P. infestans* isolate Pi-60; 10-µl droplets of zoospore suspension were pipetted on three leaves per plant. Five days later, *P. infestans* aggressiveness was observed. Left panel: control plants (Pi-60); right panel: *Methylobacterium* sp. 2A-inoculated plants (I+Pi-60).
