# Supplementary material for: Methylobacterium sp. 2A Is a Plant Growth-Promoting Rhizobacteria That Has the Potential to Improve Potato Crop Yield Under Adverse Conditions
Source: Front Plant Sci. 2020 Feb 14;11:71. doi: 10.3389/fpls.2020.00071 (PMC7038796; doi:10.3389/fpls.2020.00071)
Supplement: Supplementary file 3 [file DataSheet_3.docx]

**
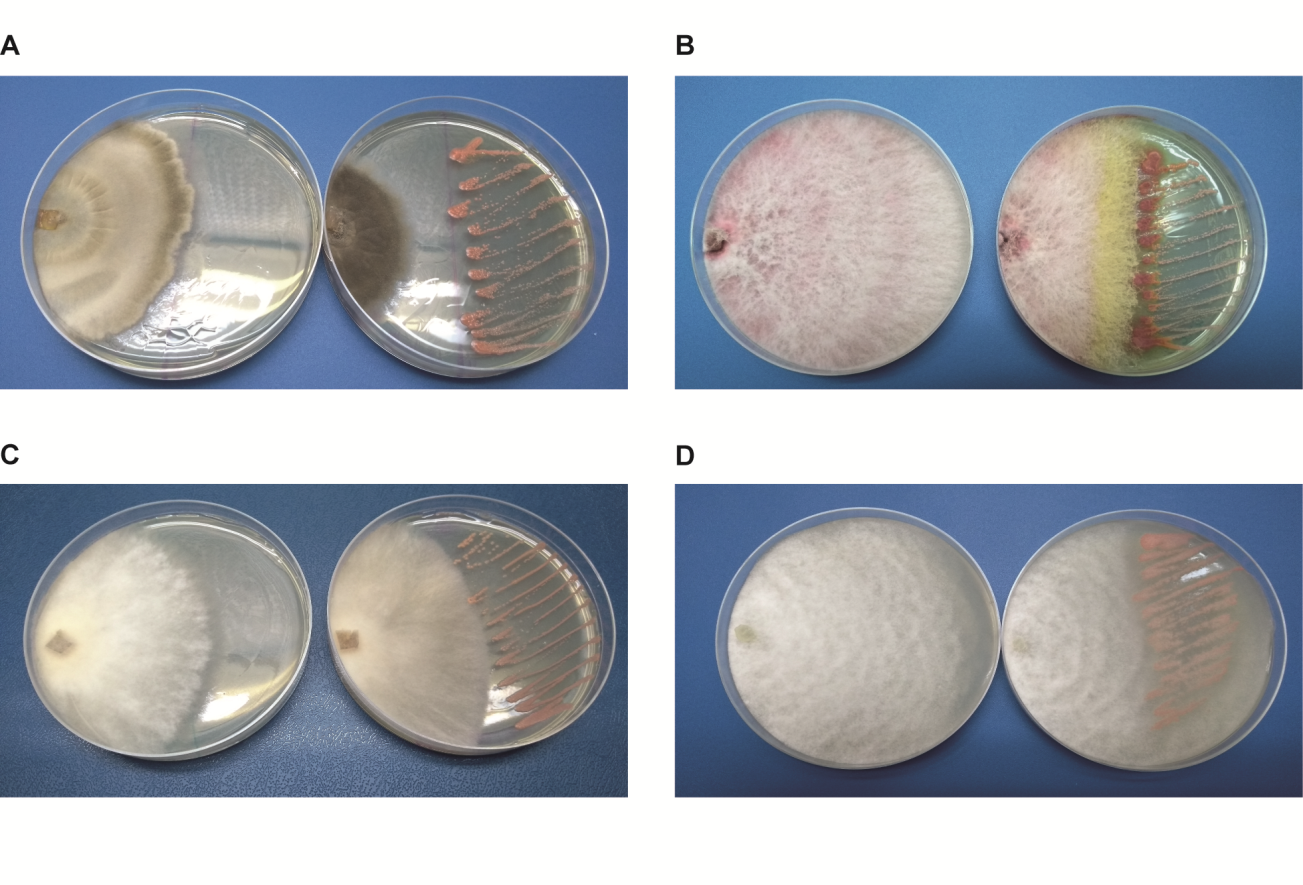
**

**Figure S3. Illustrative images showing *Methylobacterium* sp. 2A antagonistic effects against different plant pathogens in dual assays.** Images correspond to **(A)** *B. cinerea,* **(B)** *F. graminearum*, **(C)** *R. solani* and **(D)** *P. dissotocum.* Left plate of each image: pathogen; right plate: dual culture (pathogen + *Methylobacterium* sp. 2A).
