# Supplementary material for: Methylobacterium sp. 2A Is a Plant Growth-Promoting Rhizobacteria That Has the Potential to Improve Potato Crop Yield Under Adverse Conditions
Source: Front Plant Sci. 2020 Feb 14;11:71. doi: 10.3389/fpls.2020.00071 (PMC7038796; doi:10.3389/fpls.2020.00071)
Supplement: Supplementary file 4 [file DataSheet_4.docx]

**
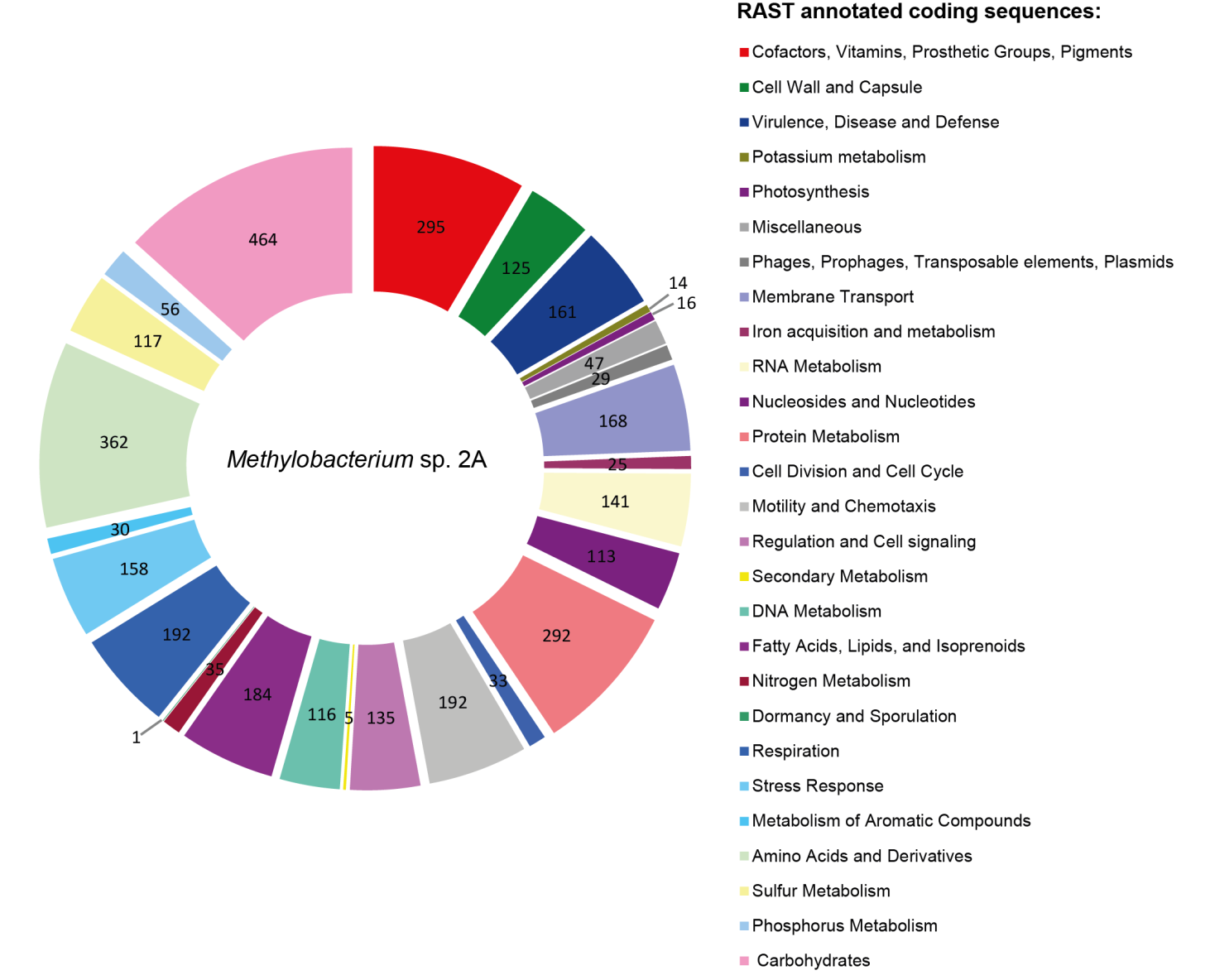
**

**Figure S4. Summary of subsystems identified by RAST of all annotated CDS.** Number of genes are indicated in all categories.
