# Supplementary material for: Methylobacterium sp. 2A Is a Plant Growth-Promoting Rhizobacteria That Has the Potential to Improve Potato Crop Yield Under Adverse Conditions
Source: Front Plant Sci. 2020 Feb 14;11:71. doi: 10.3389/fpls.2020.00071 (PMC7038796; doi:10.3389/fpls.2020.00071)
Supplement: Supplementary file 5 [file Table_1.docx]

**Table S1. Summary of primers used in this article. Primers used for 16S rDNA amplification, and those used for expression analysis of PR-1b, PAL1 and EF-1α genes.** Accession numbers of the sequences (https://www.ncbi.nlm.nih.gov/nucleotide) are included.

| Gene/Sequence | Name used | Accession number | Primers (5´-3´) |
| --- | --- | --- | --- |
| 16S ribosomal DNA | 16S rDNA | MG818293.1 | fD1: AGAGTTTGATCCTGGCTCAG  rP2: ACGGCTACCTTGTTACGACTT |
| Pathogenesis related protein 1b | PR-1b | AY050221 | StPR-1bFw: GCCCAAAATTCACCCCAAGAC  StPR-1bRv: CTGCACCGGAATGAATCAAGT |
| Phenylalanine ammonia-lyase 1 | PAL1 | KC631948.1 | PAL1-Fw: GGTGTTACTACTGGATTTGGTGC  PAL1-Rv: CCTAACAAGCATAGCTGCCC |
| Elongation factor 1 alpha | EF-1α | AB061263.1 | EF1α-Fw: TGAGGCAAACTGTTGCTGTC  EF1α-Rv: TGGAAACACCAGCATCACAC |
