# Supplementary material for: Methylobacterium sp. 2A Is a Plant Growth-Promoting Rhizobacteria That Has the Potential to Improve Potato Crop Yield Under Adverse Conditions
Source: Front Plant Sci. 2020 Feb 14;11:71. doi: 10.3389/fpls.2020.00071 (PMC7038796; doi:10.3389/fpls.2020.00071)
Supplement: Supplementary file 6 [file Table_2.docx]

| **Characteristic** | **1** | **2** | **3** | **4** | **5** | **6** | **7** |
| --- | --- | --- | --- | --- | --- | --- | --- |
| **Described in** | This study | Madhaiyan et al. 2007 | Ito and Iizuka, 1971 | Madhaiyan and Poonguzhali 2014 | Knief et al. 2012 | Kato et al. 2008 | Madhaiyan et al. 2009 |
| **Isolation source** | Roots of potato | Stem tissues of rice | Rice grains | Leaf of bamboo | Leaf of Arabidopsis | Water | Leaf of rice |
| **Morphology** |  |  |  |  |  |  |  |
| Colony pigmentation | Pink to red | Pink to red | Pink to red | Pink to red | Pale pink | Pink | Pink to red |
| Cell length (µm) | 1.6 - 2.8 | 2.1 - 2.8 | 1.4 - 2.5 | 1.3 - 2.5 | 2.4 - 12.2 | 1.3 - 2.1 | 1.8 - 2.7 |
| Cell width (µm) | 0.2 - 0.4 | 0.6 - 0.8 | 0.6 - 0.8 | 0.4 - 0.7 | 1.2 - 2.1 | 2.0 - 5.1 | 0.63 - 0.64 |
| **Oxidase** | Positive | Positive | Positive | Positive | Positive | Positive | Positive |
| **Catalase** | Positive | Positive | ND | Positive | Positive | Positive | Positive |
| **Growth conditions** |  |  |  |  |  |  |  |
| Growth temperature (°C) |  |  |  |  |  |  |  |
| Range | 20 - 30 | 20 - 30 | ND | 20 - 37 | 4 - 28 | 15 - 35 | 20 - 30 |
| Optimum | 28 | 28 | 20 - 32 | 28 | ND | 30 | 28 |
| pH range | 5.0 - 7.0 | 5.0 - 10.0 | 6 - 8 | 5.0 - 9.0 | 5.5 - 7.6 | ND | 5.0 - 9.0 |

**Table S2. Differential characteristics of *Methylobacterium* sp. 2A and its closest phylogenetic neighbors.** Strains: 1, 2A; 2, *M. oryzae* CBMB20T; 3, *M. radiotolerans* IAM 12098T; 4, *M. phyllostachyos* BL47T; 5, *M. longum* DSM 23933; 6, *M. tardum* RB677; 7, *M. phyllosphaerae* CBMB27. ND, no data.
