# Supplementary material for: Methylobacterium sp. 2A Is a Plant Growth-Promoting Rhizobacteria That Has the Potential to Improve Potato Crop Yield Under Adverse Conditions
Source: Front Plant Sci. 2020 Feb 14;11:71. doi: 10.3389/fpls.2020.00071 (PMC7038796; doi:10.3389/fpls.2020.00071)
Supplement: Supplementary file 7 [file Table_3.docx]

| **Rank** | **Name** | **Strain** | **Pairwise Similarity (%)** | **Mismatch/ Total nt** | **Completeness (%)** | **Accession** |
| --- | --- | --- | --- | --- | --- | --- |
| 1 | *M. longum* | 440 | 99,10 | 11/1218 | 100 | FN868949 |
| 2 | *M. phyllostachyos* | BL47 | 99,10 | 11/1218 | 100 | jgi.1071174 |
| 3 | *M. radiotolerans* | JCM 2831 | 98,85 | 14/1218 | 100 | CP001001 |
| 4 | *M. phyllosphaerae* | CBMB27 | 98,77 | 15/1218 | 100 | CP015367 |
| 5 | *M. tardum* | RB677 | 98,77 | 15/1218 | 99,86 | AB252208 |
| 6 | *M. oryzae* | CBMB20 | 98,60 | 17/1218 | 100 | CP003811 |
| 7 | *M. fujisawaense* | DSM 5686 | 98,52 | 18/1218 | 99,86 | AB175634 |
| 8 | *M. pseudosasicola* | BL36 | 98,11 | 23/1218 | 100 | jgi.1071178 |
| 9 | *M. mesophilicum* | JCM 2829 | 98,03 | 24/1218 | 100 | D32225 |
| 10 | *M. brachiatum* | B0021 | 98,03 | 24/1218 | 99,86 | AB175649 |
| 11 | *M. trifolii* | TA73 | 97,37 | 32/1218 | 95,95 | FR847848 |
| 12 | *M. gregans* | 002-074 | 97,36 | 32/1214 | 99,86 | AB252200 |
| 13 | *M. hispanicum* | GP34 | 97,21 | 34/1217 | 99,57 | AJ635304 |
| 14 | *M. jeotgali* | S2R03-9 | 97,04 | 36/1218 | 98,08 | DQ471331 |

**Table S3.** List of sequences which shown >97% pairwise similarity with *Methylobacterium* sp. 2A on the EzBioCloud 16S database.
